# Successful Improvement of Cardiac Function in Late-Onset Dilated Cardiomyopathy Due to the *SLC25A20* c.199-10T>G Mutation

**DOI:** 10.1016/j.jaccas.2025.104011

**Published:** 2025-08-13

**Authors:** Huan Thanh Nguyen, Phong Hoang Ninh

**Affiliations:** a Department of Geriatrics and Gerontology, University of Medicine and Pharmacy at Ho Chi Minh City, Ho Chi Minh City, Vietnam; b Department of Cardiology, Thong Nhat Hospital, Ho Chi Minh City, Vietnam

**Keywords:** carnitine-acylcarnitine translocase, dilated cardiomyopathy, *SLC25A20*

## Abstract

**Background:**

Cardiomyopathy due to *SLC25A20* mutations typically manifests in the neonatal period, characterized by rapid progression and high mortality.

**Case Summary:**

A 62-year-old man presented with late-onset dilated cardiomyopathy (DCM) associated with a heterozygous *SLC25A20* c.199-10T>G mutation. Comprehensive evaluation, including biochemical testing, echocardiography, cardiac magnetic resonance imaging, and invasive coronary angiography, ruled out other potential etiologies. The patient received guideline-directed medical therapy in conjunction with cardiac resynchronization therapy, leading to significant clinical and functional improvement.

**Discussion:**

To our knowledge, this represents the oldest reported case of *SLC25A20*-related DCM. This case highlights the clinical presentation, multimodal imaging findings, and therapeutic response in late-onset *SLC25A20*-related cardiomyopathy.

**Take-Home Messages:**

Although rare, *SLC25A20* mutations can contribute to late-onset DCM. Optimized heart failure management, including multimodal therapeutic strategies, may improve cardiac function in patients with *SLC25A20*-related DCM.

## Graphical Abstract

**Figure:**
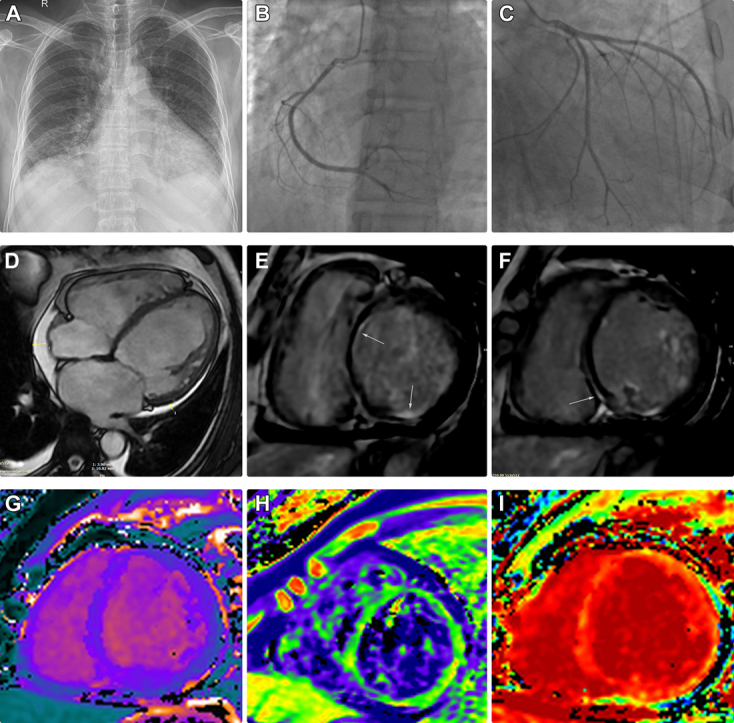

## History of Presentation

**Figure 1:**
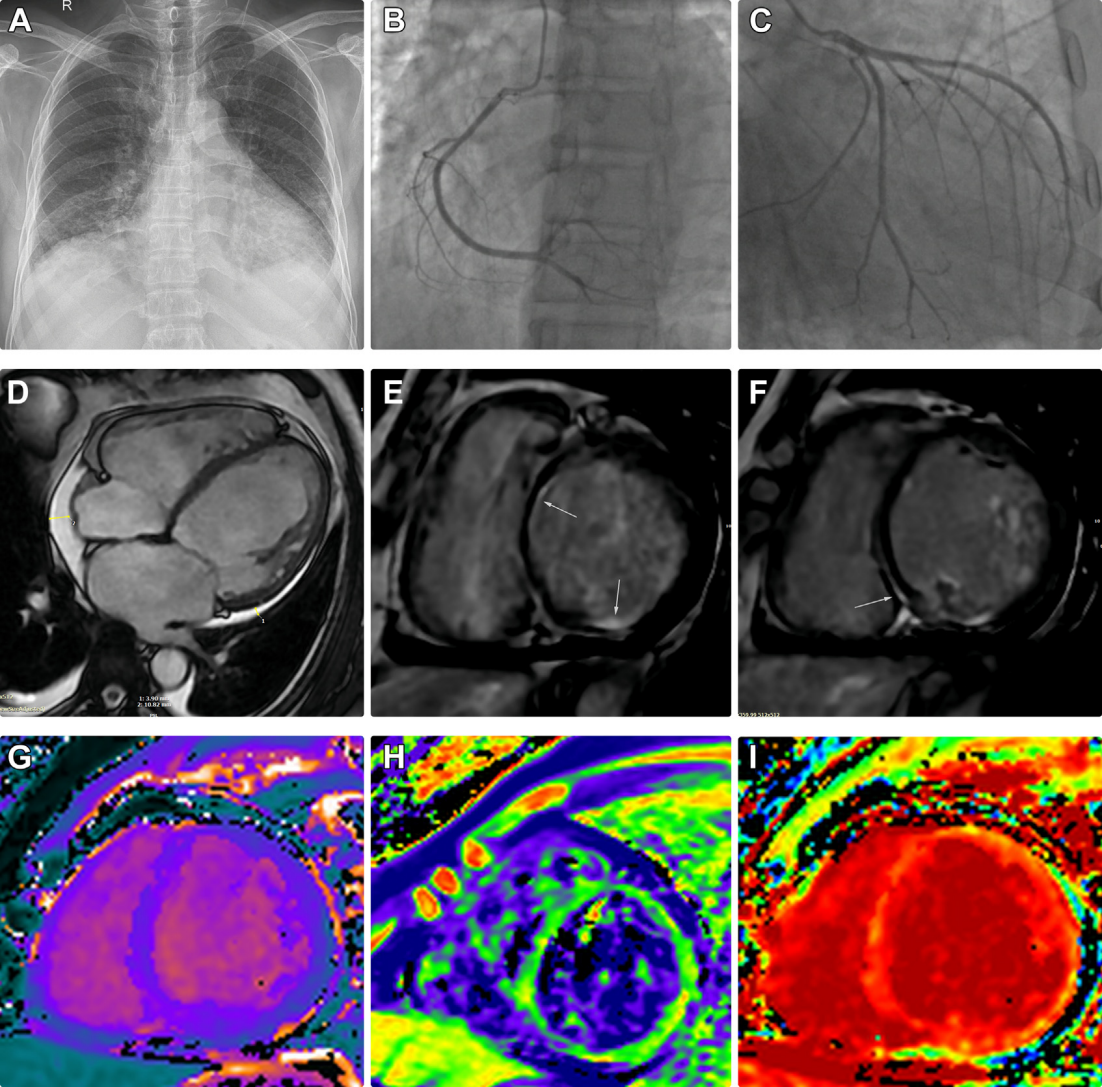
Chest X-Ray, Coronary Angiography, and Cardiac Magnetic Resonance Imaging Findings (A) Posteroanterior chest X-ray demonstrating an increased cardiothoracic ratio (>0.6). (B and C) Invasive coronary angiography revealing nonsignificant stenosis in the right and left coronary arteries. (D-I) Cardiac magnetic resonance imaging findings. (D) Four-chamber view showing dilation of all 4 cardiac chambers and a small pericardial effusion. (E and F) Late gadolinium enhancement in the subendocardial region of the interventricular septum and basal-to-mid inferior wall, with midmyocardial enhancement in the midseptal region. The total fibrosis burden was 9 g, accounting for 7% of the total left ventricular mass. (G) Increased native T1 time (1,010 ms). (H) Increased native T2 time (63 ms). (I) Increased extracellular volume fraction (45%).

A 62-year-old man presented with progressive exertional dyspnea over the past month, accompanied by episodes of paroxysmal nocturnal dyspnea. On the day of admission, he developed significant dyspnea without fever, mild chest discomfort, and bilateral lower limb edema. Chest radiography revealed cardiomegaly (Figure 1A). Electrocardiography showed sinus rhythm with complete left bundle branch block. High-sensitivity troponin T and N-terminal pro–B-type natriuretic peptide levels at admission were 35 ng/L and 5,174 pg/mL, respectively. Transthoracic echocardiography demonstrated 4-chamber dilation, global left ventricular hypokinesis, and a left ventricular ejection fraction (LVEF) of 32%.

**Take-Home Messages**

• Although rare, *SLC25A20* mutations can lead to late-onset DCM.
• Complying with optimal management strategies through multimodal approaches can improve cardiac function in patients with late-onset *SLC25A20*-related DCM.

This case study was approved by the Ethics Committee of our hospital. The authors confirm that written consent for submission and publication of this case report including images and associated text has been obtained from the patient's next of kin in line with COPE guidance.

## Medical History

A transthoracic echocardiography performed 2 years earlier during a routine health examination revealed an LVEF of 53%, mild hypokinesis of the interventricular septum, and no chamber dilation. The patient was prescribed an angiotensin II receptor blocker and dapagliflozin; however, compliance with the treatment regimen was suboptimal.

## Differential Diagnosis

The differential diagnosis of cardiac dilation and reduced LVEF includes multiple etiologies. In older adults, coronary artery disease is the most common cause. Additional considerations include inflammatory, infectious, toxic, metabolic, infiltrative, autoimmune, arrhythmic, and genetic disorders.

## Investigations

**Figure 2:**
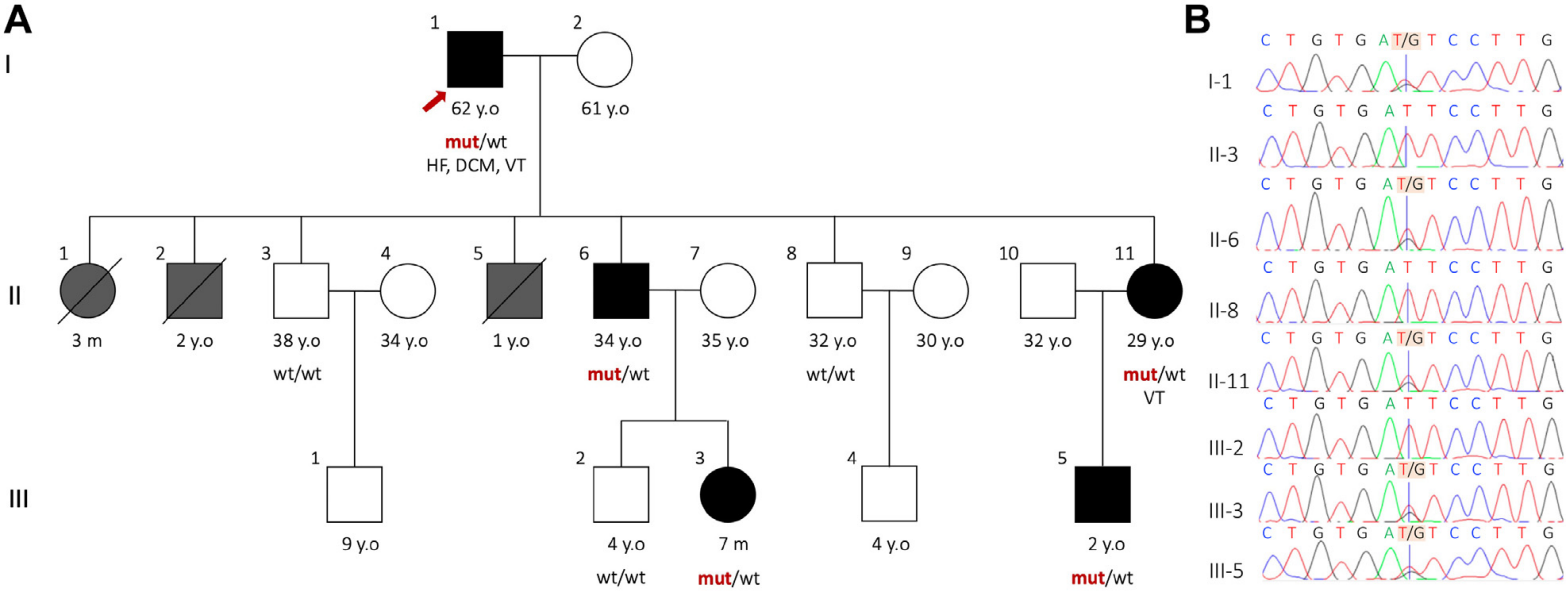
Genetic Findings and Family Pedigree (A) Family pedigree. The patient (indicated by an arrow) had 7 offspring, 3 of whom died in the neonatal period. (B) Sanger sequencing of the patient and family members identified a heterozygous intronic mutation in the *SLC25A20* gene (NM_000387.6: c.199-10T>G). DCM = dilated cardiomyopathy; HF = heart failure; m = months; mut = mutation; VT = ventricular tachycardia; wt = wild-type; y.o. = years old. Pedigree symbols: squares for males, circles for females, diagonal lines represent deceased, black-filled symbols denote affected individuals, gray-filled symbols for uncertain cardiac phenotype, and white-filled symbols denotes healthy individuals.

Invasive coronary angiography revealed a 30% stenosis in the left anterior descending artery (Figures 1B and 1C). Cardiac magnetic resonance imaging demonstrated 4-chamber dilation, late gadolinium enhancement in multiple myocardial regions, and elevated native T1, native T2, and extracellular volume fraction (Figures 1D to 1I). Autoimmune, endocrine, hepatic, and renal function tests were unremarkable. Family history revealed that 3 of the patient’s children had died in the neonatal period from unknown causes (Figure 2B). Genetic analysis identified a heterozygous intronic *SLC25A20* c.199-10T>G mutation (Figure 2A) in both the patient and his 2 surviving offspring. Despite this mutation, serum total carnitine, free carnitine, and acylcarnitine levels were within normal limits.

## Management

**Figure 3:**
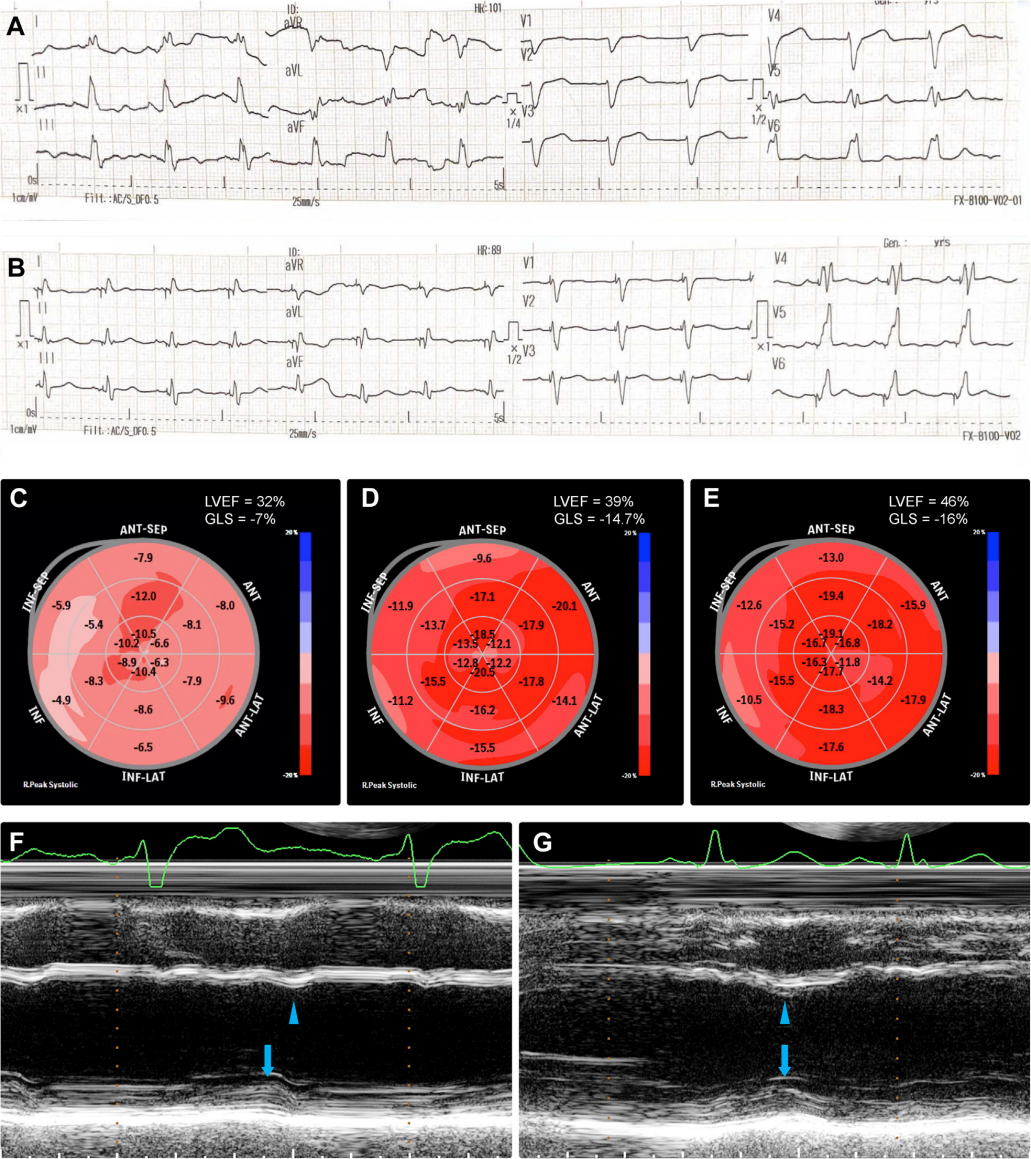
Imaging-Based Assessment of Heart Failure Treatment Response (A and B) 12-lead electrocardiogram (ECG) before and after cardiac resynchronization therapy with defibrillator (CRT-D) implantation. Pre-CRT-D implantation, ECG demonstrated complete left bundle branch block, whereas after CRT-D implantation, ECG showed reduced QRS duration. (C to E) Speckle-tracking echocardiography at admission, after 2 months of guideline-directed medical therapy, and after CRT-D implantation demonstrated improvements in left ventricular ejection fraction from 32% to 39% to 46% and in global longitudinal strain from –7% to –14.7% to –16%. (F and G) M-mode echocardiography assessing left ventricular ejection fraction using the Teichholz method showed systolic dyssynchrony between the interventricular septum (arrowhead) and posterior left ventricular wall (arrow) before CRT-D implantation, with restoration of synchrony after CRT-D implantation.

The patient received guideline-directed medical therapy for heart failure, including valsartan/sacubitril 50 mg twice daily, spironolactone 50 mg once daily, dapagliflozin 10 mg once daily, and furosemide 40 mg once daily. Within 1 week, lower limb edema had resolved and paroxysmal nocturnal dyspnea had abated. Bisoprolol 2.5 mg once daily was introduced, and furosemide was gradually tapered. After 2 months of optimized medical therapy, the patient demonstrated improved exercise tolerance, with no recurrence of dyspnea, cough, or edema. He subsequently underwent cardiac resynchronization therapy with defibrillator (CRT-D) implantation. Postimplantation, electrocardiography revealed a reduction in QRS duration (Figures 3A and 3B), whereas speckle-tracking echocardiography demonstrated improvements in global longitudinal strain and LVEF (Figures 3C to 3E). CRT-D also enhanced ventricular synchrony (Figures 3F and 3G). The patient was discharged on quadruple therapy, including valsartan/sacubitril 100 mg twice daily, spironolactone 50 mg once daily, dapagliflozin 10 mg once daily, and bisoprolol 5 mg once daily.

## Outcome and Follow-Up

At a 3-month follow-up visit after CRT-D implantation, the patient remained asymptomatic, was able to climb 3 flights of stairs without dyspnea, and remained free of edema and chest pain.

## Discussion

Carnitine-acylcarnitine translocase (CACT) deficiency is a rare disorder of long-chain fatty acid transport into mitochondria, a process dependent on carnitine. This autosomal recessive condition is caused by mutations in the *SLC25A20* gene, which encodes the CACT enzyme responsible for transporting acylcarnitines across the mitochondrial inner membrane. This function is essential for fatty acid oxidation, a key metabolic pathway for energy production.^1^ Impaired CACT activity leads to disrupted energy metabolism and cellular damage, particularly in high-energy–demanding tissues such as the myocardium and skeletal muscle. A recent study demonstrated that the *SLC25A20* c.199-10T>G variant results in reduced protein stability and increased aggregation, potentially disrupting mitochondrial function.^2^

To date, *SLC25A20*-related cardiomyopathy has been reported almost exclusively in neonates, characterized by rapid progression and high mortality. Here, we report the first known case of an older adult diagnosed with dilated cardiomyopathy (DCM) harboring the *SLC25A20* c.199-10T>G variant. Notably, this patient exhibited significant clinical and functional improvement after optimal medical therapy and CRT-D. Through comprehensive genetic and clinical evaluation, this case provides the first characterization of late-onset DCM associated with an *SLC25A20* mutation.

Several mechanisms may explain the delayed disease onset in this patient. First, variable genetic penetrance may modulate the phenotypic expression of DCM.^3^ Second, residual CACT protein function may partially preserve fatty acid metabolism, thereby delaying disease onset.^4^ Third, metabolic flexibility in adults, including greater reliance on glucose metabolism and ketone body utilization, may compensate for impaired fatty acid oxidation, mitigating early disease progression. In contrast, fatty acid oxidation is the predominant energy source in infancy, rendering defects in this pathway more detrimental.^5^^,^^6^ Finally, progressive mitochondrial dysfunction and accumulated oxidative stress over time may ultimately impair cardiac energy homeostasis, contributing to the development of late-onset DCM.^7^

## Conclusions

This report provides the first description of the clinical characteristics, imaging findings, and successful therapeutic intervention leading to improved cardiac function in an older adult with an *SLC25A20* mutation. Further research is needed to elucidate the pathophysiological mechanisms underlying the late-onset presentation of this genetic variant.

## Funding Support and Author Disclosures

The authors have reported that they have no relationships relevant to the contents of this paper to disclose.
